# Crosstalk Between Oxidative Stress, Protein Glycation, and Extracellular Matrix Remodeling in the Skin of Rats with Type 1 Diabetes: Does Insulin Administration Improve Skin Homeostasis?

**DOI:** 10.3390/antiox15060726

**Published:** 2026-06-07

**Authors:** Natalia Dorf, Edyta Gołaś, Cezary Pawlukianiec, Małgorzata Żendzian-Piotrowska, Anna Zalewska, Mateusz Maciejczyk

**Affiliations:** 1 Independent Laboratory of Cosmetology, Medical University of Bialystok, 3 Akademicka Street, 15-267 Bialystok, Poland; natalia.dorf@umb.edu.pl; 2 Students’ Scientific Club “Biochemistry of Civilization Diseases” at the Department of Hygiene, Epidemiology and Ergonomics, Medical University of Bialystok, 2c Mickiewicza Street, 15-233 Bialystok, Poland; edyta.golas@sd.umb.edu.pl (E.G.); cezary.pawlukianiec@gmail.com (C.P.); 3 Department of Hygiene, Epidemiology and Ergonomics, Medical University of Bialystok, 2c Mickiewicza Street, 15-233 Bialystok, Poland; mzpiotrowska@gmail.com; 4 Independent Laboratory of Experimental Dentistry, Medical University of Bialystok, 24a M. Sklodowskiej-Curie Street, 15-274 Bialystok, Poland; azalewska426@gmail.com

**Keywords:** diabetes, skin in diabetes, oxidative stress, glycoxidation

## Abstract

The exact mechanisms of skin involvement in type 1 diabetes (DM1) remain poorly understood. This study aimed to evaluate the relationship between antioxidants, oxidative stress, protein glycation, and glycoxidation, as well as matrix metalloproteinase (MMP) activity, in the skin of rats with DM1, while investigating whether insulin administration improves skin homeostasis. Male Wistar rats were assigned to three groups: control, diabetes, and diabetes treated with insulin. Significantly higher expression of GSH (gluthatione) and GSH-Px (glutathione peroxidase), elevated levels of AGE (Advanced Glycation End products), DT (dityrosine), KN (kynurenine), NFKN (N-formylkynurenine) and ONOO- (peroxynitrite), as well as increased activity of GLU (β-D-glucuronidase), NADPH oxidase (NOX) and MMP-1, -2, -3, -7, -9, -11 and -13 were observed in the skin of rats with DM1. Insulin treatment normalizes the skin’s antioxidant barrier and eliminates oxidative stress. It also reduces the intensity of protein glycation and glycoxidation, though not to the levels observed in the control group. Summarizing, in diabetic skin there is a complex interaction between the thiol antioxidant barrier, oxidative damage, protein glycation and glycoxidation as well as MMP expression. Insulin restores physiological balance in skin cells; however, glycation and ECM remodeling are still more pronounced than in healthy skin.

## 1. Introduction

The prevalence of diabetes mellitus has increased over the past few decades and has become a significant global public health problem in terms of both mortality and morbidity [1]. Type 1 diabetes (DM1) constitutes about 5–10% of all diabetes cases [2]. DM1 is classically defined by autoimmune destruction of the insulin-producing pancreatic β-cell islets, leading to severe insulin deficiency [3]. Genetic and environmental factors, especially infectious agents, are potential triggers of the autoimmune process [4].

DM1 is a metabolic disorder characterized by chronic hyperglycemia, which contributes to abnormalities in carbohydrate, lipid, and protein metabolism. If left untreated, it leads to numerous complications at the microvascular (retinopathy, nephropathy, and neuropathy) and macrovascular (heart attack, hypertension, hyperlipidemia, and stroke events) levels [5]. Complications of DM1 can also involve the skin. Skin manifestations of DM1 include foot ulcers, diabetic dermopathy, acanthosis nigricans, bullosis diabeticorum, scleredema diabeticorum, and necrobiosis lipoidica [5,6]. Recently, oxidative stress (OS) has been recognized as a key factor in the development and progression of DM1. An imbalance between the production of reactive oxygen species (ROS) and the body’s defense mechanisms against oxidation modulates several intracellular pathways, leading to impaired β-cell function [7,8,9,10]. DNA base modification, lipid peroxidation, and protein glycoxidation are well-documented ROS-mediated pathologies which, along with elevated blood glucose levels, contribute to increased inflammation [8,11,12,13]. OS disrupts antioxidant defenses against ROS and leads to cell death by intensifying key executioner enzymes in apoptosis [14,15,16]. Studies confirm that advanced glycation end products (AGEs) are also key factors in the progression of DM1 [10,17]. These non-enzymatically derived glycation products interact with specific receptors for advanced glycation end products (RAGE) [18]. AGE/RAGE interaction increases ROS production and triggers a variety of downstream effectors, including mitogen-activated protein kinase (MAPK), p38, and protein kinase C (PKC) [13]. These kinases downregulate insulin receptor expression, leading to defective insulin receptor signaling [17,19]. There are reports of direct cross-linking between AGEs and extracellular matrix (ECM) proteins in skin, such as collagen and elastin, making them stiff and less flexible [19,20]. Excessive ROS production increases the activity of matrix metalloproteinases (MMPs), a family of enzymes capable of degrading ECM components. It has been shown that expression of metalloproteinase 1 (MMP-1), metalloproteinase 3 (MMP-3), metalloproteinase 9 (MMP-9), and metalloproteinase 12 (MMP-12) increases in DM1, leading to collagen fragmentation, disturbances in ECM remodeling, and inflammation [13,21,22]. However, the effect of insulin therapy on ECM remodeling remains unknown.

Diabetes is a common condition, yet reliable research into the effects of hyperglycemia on skin physiology remains limited. Most studies focus on systemic complications and the well-known organ failures associated with DM1, leaving a significant gap in research on skin complications. Over the past decades, several hypotheses have been proposed to explain skin involvement in DM1; however, there remains a lack of comprehensive research on the interactions among OS, protein glycation, and ECM remodeling, and on their exact contributions to the skin complications of DM1. Finally, there is also a lack of studies assessing the impact of insulin therapy on skin parameters, including oxidative damage and ECM remodeling. It is suggested that insulin’s effect on the skin is due to a systemic reduction in blood glucose levels. However, the effect of insulin on skin cells in DM1 remains unknown. This study aimed to evaluate the relationship between enzymatic and non-enzymatic antioxidants, oxidative damage to proteins and lipids, protein glycation and glycoxidation and MMP activity in the skin of rats with DM1 and to investigate whether insulin administration improves skin homeostasis.

## 2. Materials and Methods

### 2.1. Animal Treatment

The study was approved by Ethical Committee for Animal Experiments at the University of Warmia and Mazury in Olsztyn, Poland (approval no.: 53/2022) in accordance with European Directive 2010/63/EU for animal experiments and “Guide for the Care and Use of Laboratory Animals” by the National Academy of Sciences [23]. The experimental study was conducted at the Experimental Medicine Centre (EMC) of the Medical University of Bialystok. Healthy male Wistar rats weighing 67–72 g were housed under standard conditions (20–24 °C, in a cycle 12 h light/12 h dark), having constant visual contact with each other. For a period of 12 weeks, the rats of all groups of the study were fed a diet by V1534–300 ssniff Spezialdiaten GmbH (Soest, Germany), containing 67% carbohydrate, 24 kcal% protein and 9 kcal% fat. Throughout the entire study, the rats had unrestricted access to water and food. After 4 weeks of acclimatization, rats assigned to the study were divided randomly into three groups: control (C, 10 rats), DM1 group (DM1, 10 rats) and DM1 group with intraperitoneal insulin injection (DM1+Insulin, 10 rats).

**Figure 1:**
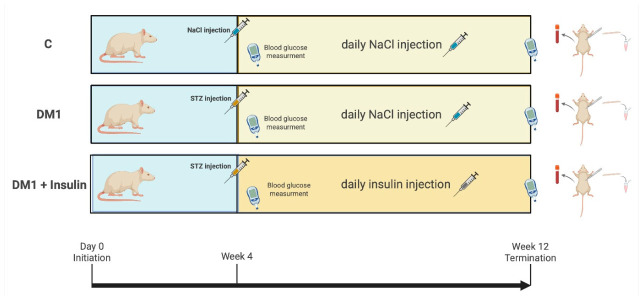
Experimental groups: C: control group; DM1: diabetic rat group; DM1+insulin: insulin-treated diabetic rat group; NaCl: sodium chloride; STZ: streptozotocin. Created in BioRender. Maciejczyk, M. (2026) https://BioRender.com/6pnz61y (accessed on 27 April 2026).

To induce DM1 in the 4th week of the experiment, rats of two groups had a single intraperitoneal injection of streptozotocin (STZ) at a dose of 60 mg/kg body weight, while control rats received an equivalent volume of citrate buffer, pH 4.5 (placebo), which served as the solvent for STZ [24,25]. To confirm DM1, fasting plasma glucose was measured using a standard procedure with a glucometer (Accu-Chek; Bayer, Leverkusen, Germany). Blood was drawn from tail vein 48 h after STZ administration [26]. All rats receiving STZ had blood glucose levels > 250 mg/dL, which confirmed the diagnosis of DM1. Next, rats in the DM1+Insulin group were given daily intraperitoneal insulin at a dose of 0.5 IU/kg for the next 8 weeks, while rats in the other two groups received intraperitoneal injections of physiological saline [27]. This particular dose is commonly used to achieve a state close to normoglycemia without causing adverse effects in rats, particularly severe and fatal hypoglycemia [28,29,30]. After 8 weeks, all rats were anesthetized by intraperitoneal injection of sodium pentobarbital (40 mg/kg of body weight) for tissue collection [31]. The rats were euthanized using rapid exsanguination by drawing blood from the abdominal artery. To obtain plasma, blood samples were collected into tubes containing a buffered trisodium citrate solution. After centrifugation (3000× *g*, 4 °C, 10 min), the plasma was stored at −80 °C for further biochemical analysis. Next, an experienced technician shaved the dorsal skin and collected skin samples. The tissues were pre-cleaned (to remove blood, fascia, or fat), pre-cooled in liquid nitrogen and then stored at −80 °C until analysis. Skin samples were cut into small pieces and homogenized in a 1:9 ratio of phosphate-buffered saline (PBS; 0.1 M, pH 7.4) containing protease inhibitors [32]. Next, the skin homogenates were subjected to sonication on ice (1800 J per sample, 2 × 15 s; sonifier UP 400S, Hielscher, Teltow, Germany), and then centrifuged at 10,000× *g* for 10 min at 4 °C. The resulting supernatants were stored at −80 °C for further experiments (Figure 1) [33].

### 2.2. Biochemical Determinations

All reagents (unless otherwise specified) were purchased from Sigma-Aldrich (Nümbrecht, Germany/Saint Louis, MO, USA): 1,1,3,3-tetraethoxypropane (cat. no. T9889), 3-[(3-cholamidopropyl)dimethyl-ammonio]-1-propanesulfonate hydrate (CHAPS; cat. no. C3023), 4-aminophenylmercuric acetate (APMA; cat. no. A9563), 4-nitrophenyl 2-acetamido-2-deoxy-β-D-glucopyranoside (HEX; cat. no. N9376), 4-nitrophenyl β-D-glucuronide (GLU; cat. no. N1627), β-Nicotinamide adenine dinucleotide, reduced tetrasodium salt hydrate (NADPH, cat. no. 10107824001), 5,5’-dithiobis(2-nitrobenzoic acid) (DTNB; cat. no. D8130), acetic acid (cat. no. 27225), caspase-6 substrate III, fluorogenic (cat. no. 218763), citric acid monohydrate (cat. no. C1909), dimethyl sulfoxide (cat. no. D2650), dimethyl sulfoxide (DMSO; cat. no. D4540), disodium hydrogen phosphate (Na_2_HPO_4_; cat. no. S5136), DL-dithiothreitol (DTT; cat. no. D0632), egtazic acid (EGTA; cat. no. E3889), epinephrine (cat. no. E4375), ethylenediaminetetraaceticacid (EDTA; cat. no. E9884), ethylenediaminetetraaceticacid solution (EDTA; cat. no. E8008), ferric chloride (FeCl_3_; cat. no. 157740), glacial acetic acid (cat. no. 33209-M), glutathione reductase (cat. no. G3664), HEPES (cat. no. H3375), hydrochloric acid (HCl; cat. no. 320331), hydrogen peroxide (H_2_O_2_; cat. no. H1009), lucigenin (cat. no. S1174), magnesium chloride (MgCl_2_; cat. no. M8266), methanesulfonic acid (cat. no. 471356), MMP Substrate (cat. no. SCP0193), N-acetyl-asp-glu-val-asp-p-nitroanilide (cat. no. A2559), nicotinamide adenine dinucleotide phosphate, reduced form (NADPH; cat. no. N7505), nicotinamide adenine dinucleotide, reduced form (NADH; cat. no. N8129), nitro blue tetrazolium (cat. no. N6639), phenol (cat. no. 242322), p-nitroaniline (cat. no. 185310), potassium phosphate monobasic (KH_2_PO_4_; cat. no. P5655), reduced glutathione (GSH; cat. no. G4251), sodium acetate (cat. no. S8750), sodium azide (NaN_3_; cat. no. S2002), sodium bicarbonate (NaHCO_3_; cat. no. S5761), sodium carbonate (Na_2_CO_3_; cat. no. S7795), sodium chloride (NaCl; cat. no. S9888), sodium hydroxide (KOH; cat. no. 221465), sodium phosphate dibasic dodecahydrate (Na_2_HPO_4_ · 12H_2_O; cat. no. 04273), sodium phosphate monobasic (cat. no. S5011), sodium tetraborate (Na_2_B_4_O_7_; cat. no. 221732), streptozotocin (STZ; cat. no. S0130), sucrose (cat. no. 84097), trizma base (cat. no. T1503). Potassium chloride (KCl; cat. no. 739740421) was purchased from Pol-Aura Sp. z o.o. (Poland) and calcium chloride anhydrous (CaCl_2_; cat. no. 118748703) from Chempur (Poland). Type I reagent-grade deionized water was also utilized. The absorbance and fluorescence of the samples were measured using a 96-well microplate reader (Infinite M200 PRO microplate reader, Tecan, Männedorf, Switzerland; Biotek ELx800, BioTek Instruments, Winooski, VT, USA). The results were normalized to total protein content.

#### 2.2.1. Protein Content

Total skin protein concentration was assessed using the commercial Thermo Scientific PIERCE BCA Protein Assay kit (cat. no. 23225; Rockford, IL, USA) using the bicinchoninic acid method. The method involves the reduction of Cu^2+^ to Cu^+^ by protein in the presence of sodium potassium tartrate and the subsequent reaction of bicinchoninic acid (BCA) with copper(I) cations. The BCA-copper complex develops a purple color, the intensity of which, measured at 562 nm, is proportional to the protein concentration present in the sample [34,35,36,37].

#### 2.2.2. Apoptosis

Peroxynitrite (ONOO-) concentration was assessed according to the method described by Beckman [38], which involves the nitration of phenol (4-HPA) by ONOO- to 4-hydroxy-3-nitrophenylacetic acid (NO_2_-HPA). The reaction is catalyzed by metal ions (Fe^3+^–EDTA) or the enzyme superoxide dismutase (SOD). NO_2_-HPA is the reaction product, the concentration of which is measured spectrophotometrically at 430 nm. The amount of product formed reflects the concentration of peroxynitrite in the sample.

Caspase-3 (Cas-3) activity (EC: 3.4.22.56) was assessed by a colorimetric method using the specific substrate N-Acetyl-Asp-Glu-Val-Asp p-nitroanilide (Ac-DEVD-pNA). The amount of p-nitroaniline (pNA) released during the enzymatic reaction was determined by measuring absorbance at 405 nm, and its amount was proportional to enzyme activity [39]. Caspase-6 (Cas-6) activity (EC: 3.4.22.59) was assessed fluorimetrically. The specific substrate used in the enzymatic reaction was trifluoroacetate salt (Z-VEID-AFC) [40].

#### 2.2.3. Lysosomal Exoglycosidases

The activity of lysosomal exoglycosidases in the skin was assessed using their ability to hydrolyze synthetic substrates. 4-Nitrophenyl-β-D-glucuronide and 4-nitrophenyl-N-acetyl-β-glucosaminide were used to assess N-acetyl-β-hexosaminidase (HEX) (EC: 3.2.1.52) and β-glucuronidase (GLU) (EC: 3.2.1.31) activity, respectively. The product of these reactions is yellow 4-nitrophenol, the concentration of which is measured spectrofluorimetrically at 405 nm. The amount of the product formed reflects the HEX and GLU activity in the sample [41,42].

#### 2.2.4. Antioxidant Barrier

The concentration of reduced glutathione (GSH) was determined by a colorimetric method using a reaction with 5.5′-dithiobis-(2-nitrobenzoic acid) (DTNB) [43]. The resulting colored complex was analyzed spectrophotometrically at a wavelength of 412 nm.

Glutathione peroxidase (GSH-Px) activity (EC: 1.11.1.9) was determined colorimetrically according to the method described by Paglia and Valentine [44]. The method involved measuring the decrease in absorbance of nicotinamide adenine dinucleotide (NADPH) during its oxidation to nicotinamide adenine dinucleotide phosphate (NADP^+^). Absorbance measurements were performed at a wavelength of 340 nm. A unit of GSH-Px activity was defined as the amount of enzyme catalyzing the oxidation of 1 μmol of NADPH in one minute.

SOD activity (EC: 1.15.1.1) was determined spectrophotometrically based on the degree of inhibition of adrenaline oxidation to adrenochrome. A unit of SOD activity was defined as the amount of enzyme inhibiting adrenaline oxidation by half. The measurement was performed at a wavelength of 480 nm [45].

Catalase (CAT) activity (EC: 1.11.1.6) was determined using the method described by Hugo Aebi [46], which involves colorimetric measurement of the rate of H_2_O_2_ decomposition. Absorbance measurements were performed at a wavelength of 240 nm. A unit of activity was defined as the amount of enzyme required to decompose one nmol of hydrogen peroxide in one minute. Enzymatic reaction intensity was expressed as nmol H_2_O_2_/min/mg protein.

#### 2.2.5. Protein Glycation and Glycoxidation

AGE content in skin tissue homogenates was assessed spectrofluorimetrically. For this purpose, samples were diluted 10-fold in a sodium phosphate buffer and thoroughly mixed on a microplate shaker. AGE fluorescence emission was recorded in 200 µL samples at excitation and emission wavelengths of 350 and 440 nm [47]. Fluorescence intensity was converted to the protein content and expressed in fluorescence units (AFU)/mg protein.

The contents of dityrosine (DT), kynurenine (KN), and N-formylkynurenine (NFKN) were analyzed spectrofluorimetrically. For measurements, samples were diluted 10-fold in sodium phosphate buffer and thoroughly mixed on a microplate shaker. Specific fluorescence was measured in 200 µL samples at wavelengths of 330/415, 365/480, and 325/434, respectively [48]. Fluorescence intensity was converted to the protein content and expressed in AFU/mg protein.

NADPH oxidase (NOX) (EC 1.6.3.1) activity was determined by the luminescence assay, where lucigenin served as the electron acceptor [49]. One unit of NOX activity was defined as the quantity of enzyme required to release 1 nmol of superoxide anion per 1 min.

Nuclear Factor kappa B (NF-κB) level was determined using a sandwich ELISA method with a commercial ELISA kit (cat. no. SEB824Hu, Cloud-Clone Corp., CCC, Wuhan, China) that utilizes a specific antibody against NF-κB. The detection signal was generated using a biotin–avidin system conjugated with horseradish peroxidase (HRP). The intensity of the colorimetric reaction with 3,3′,5,5′-tetramethylbenzidine (TMB), measured at 450 nm, was proportional to the expression of NF-κB in the sample.

#### 2.2.6. ECM Remodeling

MMP activity was measured fluorimetrically. To activate the enzymes, samples were incubated with 4-aminophenylmercuric acetate (APMA) at 37 °C for 3 h for MMP-1, 1 h for metalloproteinase 2 (MMP-2) (EC: 3.4.24.24), 24 h for MMP-3, 20 min for metalloproteinase-7 (MMP-7) (EC: 3.4.24.23), 2 h for MMP-9, and 40 min for metalloproteinase 13 (MMP-13) (EC: 3.4.24). For metalloproteinase 11 (MMP-11) (EC: 3.4.24.22), samples were ready for reading without an incubation period. The substrate for the enzymatic reaction was MCA-Pro-Leu-Gly-Leu-Dpa(Dnp)-Ala-Arg-NH_2_, where the fluorophore was 7-methoxycoumarin-4-yl) acetate (MCA). Fluorescence was measured at an excitation wavelength of 325 nm and an emission wavelength of 393 nm [50].

### 2.3. Statistical Analysis

Statistical analyses were carried out using GraphPad Prism version 8.3.0 for macOS (GraphPad Software, Inc., La Jolla, CA, USA). The assumptions of normality and homogeneity of variance were verified using the Shapiro–Wilk and Levene’s tests, respectively. Quantitative variables were compared using the Kruskal–Wallis test followed by Dunn’s post hoc test. Multiplicity-adjusted *p*-values were calculated. The results are presented as the minimum, median, maximum, and percentiles. The Spearman rank correlation coefficient was used to evaluate the relationship between the assessed parameters. The level of statistical significance was set at *p* < 0.05.

## 3. Results

### 3.1. General Characteristics of Rats

**Figure 2:**
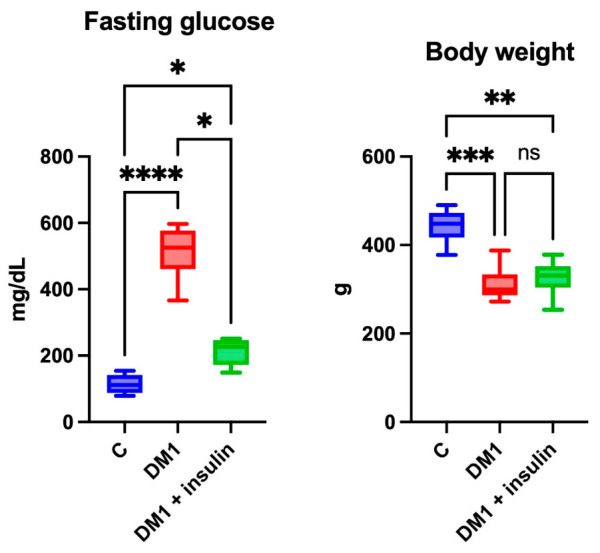
Fasting blood glucose measurement and animal body weight of C (n = 10; blue bars), DM1 (n = 10; red bars), and DM1+insulin (n = 10; green bars) rats. C: control group; DM1: diabetic rat group; DM1+insulin: insulin-treated diabetic rat group. The results are presented as the minimum, median, maximum, and percentiles. ns: not significant. * *p*  <  0.05; ** *p* < 0.01; *** *p* < 0.001; **** *p* < 0.0001.

In our experiment, DM1 was induced by intraperitoneal injection of STZ at a dose of 60 mg/kg body weight. Diabetes was diagnosed based on reference values, assuming a blood glucose concentration equal to or higher than 250 mg/dL (11.1 mmol/L), commonly used as one of the main diagnostic criteria for diabetes [47]. Blood glucose concentration in DM1 animals was significantly higher (371%) than in control animals. Glycemic concentration in insulin-treated rats was significantly lower (−57%) than in DM1 animals, but significantly higher (103%) than in control rats. The body weight of DM1 animals was significantly lower (−33%) than in the control group. The body weight of insulin-treated diabetic animals was also significantly lower (−26%) than in the control group (Figure 2).

### 3.2. The Effect of Insulin Therapy on Apoptosis-Related Biomarkers in the Skin of Diabetic Rats

**Figure 3:**
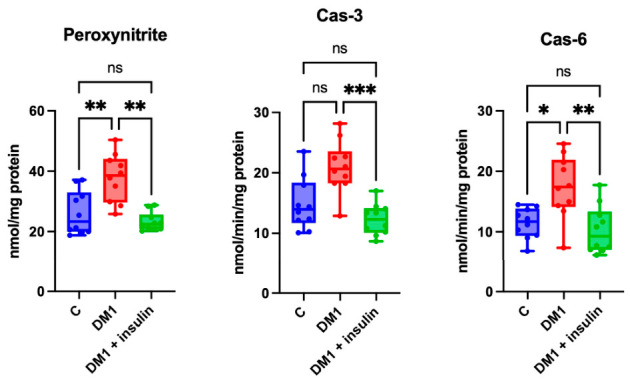
Peroxynitrite concentration, Cas-3 activity, and Cas-6 activity in skin tissue homogenates in C (n = 10; blue bars), DM1 (n = 10; red bars), and DM1+insulin (n = 10; green bars) rats. C: control group; DM1: diabetic rat group; DM1+insulin: insulin-treated diabetic rat group; Cas-3: caspase-3; Cas-6: caspase-6. The results are presented as the minimum, median, maximum, and percentiles. ns: not significant. * *p*  <  0.05; ** *p* < 0.01; *** *p* < 0.001.

We showed that the ONOO- concentration in the skin of DM1 rats was significantly higher (65%) compared to the control group. Moreover, the ONOO- concentration in the skin of insulin-treated animals was significantly lower (−42%) than in the skin of DM1 group. Cas-3 activity was significantly lower (−40%) in the skin of DM1 rats treated with insulin compared to DM1 group. Similarly, Cas-6 activity was higher (49%) in the skin of DM1 rats compared to the control group. Moreover, the activity of Cas-6 in the skin of insulin-treated rats was significantly lower (−47%) than in the DM1 group (Figure 3).

### 3.3. The Effect of Insulin Therapy on Lysosomal Exoglycosidase Activity in the Skin of Diabetic Rats

**Figure 4:**
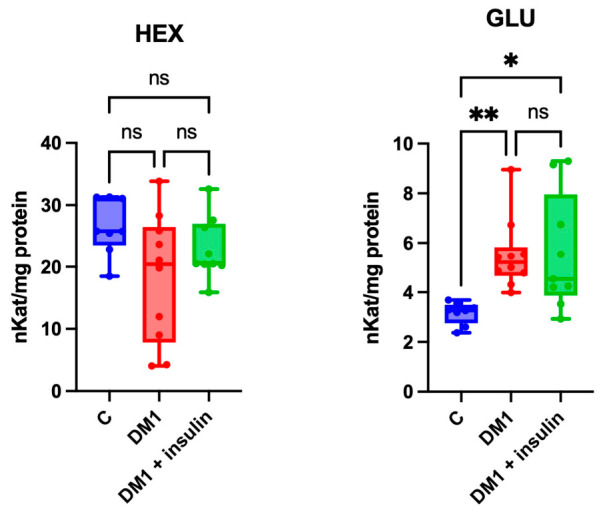
Lysosomal exoglycosidase activity in skin tissue homogenates in C (n = 10; blue bars), DM1 (n = 10; red bars), and DM1+insulin (n = 10; green bars) rats. C: control group; DM1: diabetic rat group; DM1+insulin: insulin-treated diabetic rat group; HEX: N-acetyl-beta-D-hexosaminidase; GLU: beta-glucuronidase. The results are presented as the minimum, median, maximum, and percentiles. ns: not significant. * *p*  <  0.05; ** *p* < 0.01.

GLU activity was significantly higher (59%) in the skin of DM1 rats compared to the control group. Moreover, GLU activity in the skin of insulin-treated diabetic animals was significantly higher (39%) than in the skin of the control group (Figure 4).

### 3.4. The Effect of Insulin Therapy on Antioxidant Barrier in the Skin of Diabetic Rats

**Figure 5:**
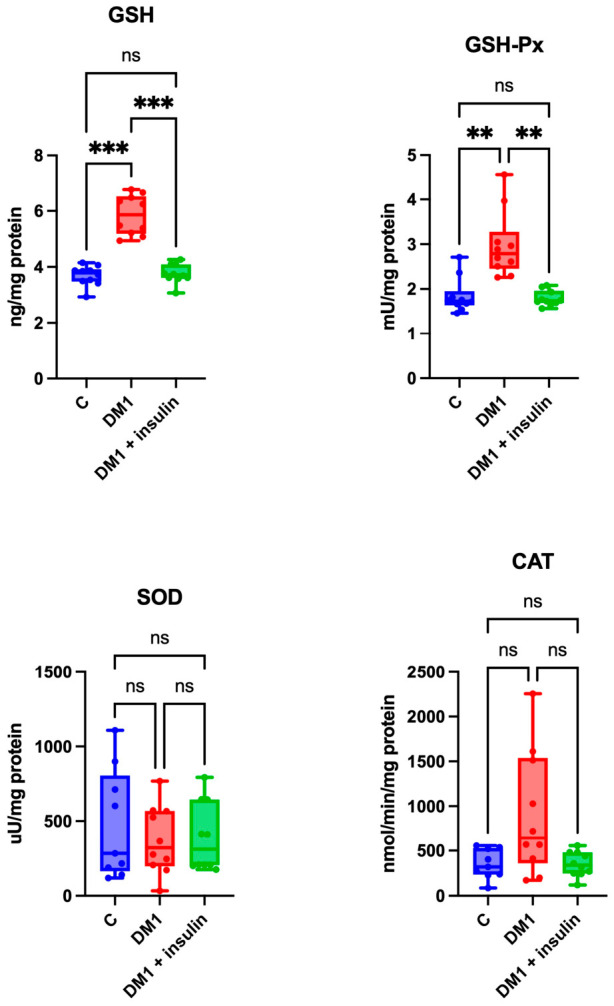
Non-enzymatic and enzymatic antioxidants in skin tissue homogenates in C (n = 10; blue bars), DM1 (n = 10; red bars), and DM1+insulin (n = 10; green bars) rats. C: control group; DM1: diabetic rat group; DM1+insulin: insulin-treated diabetic rat group; GSH: reduced glutathione; GSH-Px: glutathione peroxidase; SOD: superoxide dismutase; CAT: catalase. The results are presented as the minimum, median, maximum, and percentiles. ns: not significant. ** *p* < 0.01; *** *p* < 0.001.

GSH concentration was significantly higher (55%) in the skin of DM1 rats compared to the control group. Moreover, GSH concentration in the skin of insulin-treated diabetic rats was significantly lower (−37%) than in the skin of DM1 rats. In turn, GSH-Px activity was significantly higher (61%) in the skin of DM1 animals than in the control group. In addition, GSH-Px activity was lower (−38%) in the skin of insulin-treated diabetic rats compared to DM1 group (Figure 5).

### 3.5. The Effect of Insulin Therapy on Protein Glycation and Glycoxidation in the Skin of Diabetic Rats

**Figure 6:**
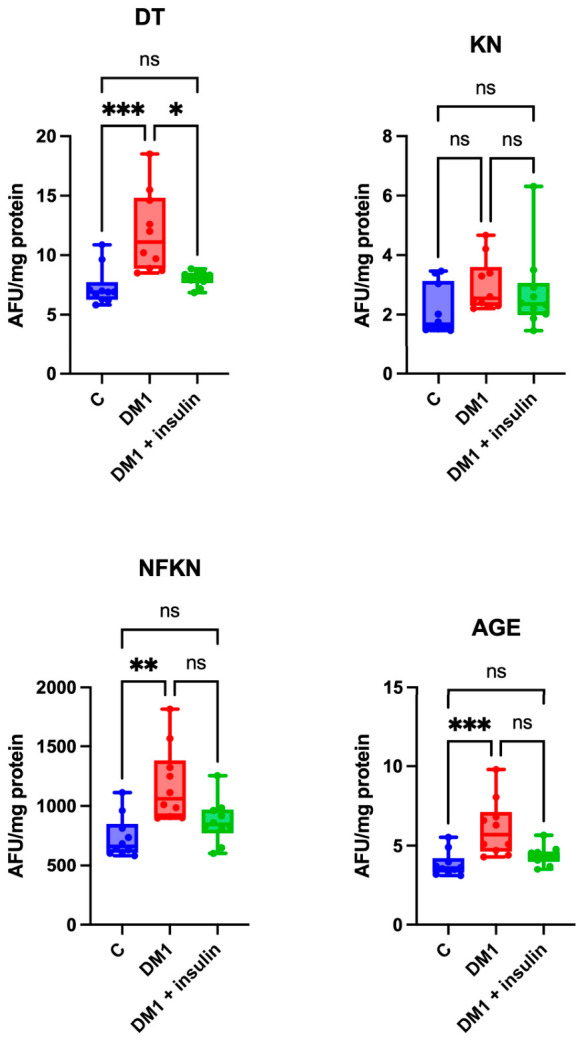
Protein glycation and glycoxidation products in C (n = 10; blue bars), DM1 (n = 10; red bars), and DM1+insulin (n = 10; green bars) rats. C: control group; DM1: diabetic rat group; DM1+insulin: insulin-treated diabetic rat group; DT: dityrosine; KN: kynurenine; NFKN: N-formylkynurenine; AGE: advanced glycation end product. The results are presented as the minimum, median, maximum, and percentiles. ns: not significant. * *p*  <  0.05; ** *p* < 0.01; *** *p* < 0.001.

DT content in the skin of DM1 rats was markedly higher (60%) compared to the skin of rats in the control group. However, the DT content in the skin of diabetic rats treated with insulin was significantly lower (−27%) than in the skin of DM1 group. Moreover, the study demonstrated that the NFKN content was significantly higher (61%) in the skin of DM1 rats compared to the control group. A similar pattern was observed for AGE, whose levels in the skin of DM1 rats were significantly higher (59%) than in the skin of rats from the control group (Figure 6).

**Figure 7:**
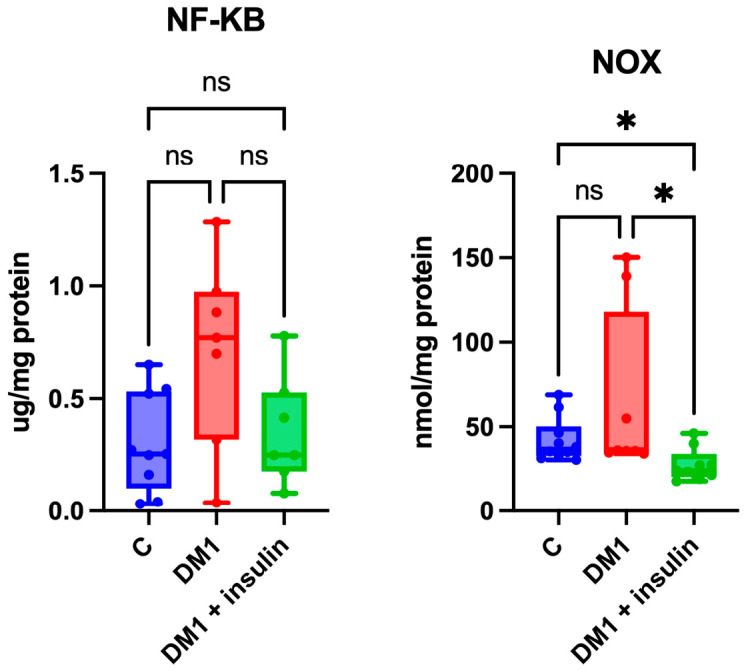
AGE downstream mediators in C (n = 10; blue bars), DM1 (n = 10; red bars), and DM1+insulin (n = 10; green bars) rats. C: control group; DM1: diabetic rat group; DM1+insulin: insulin-treated diabetic rat group; NFkB: nuclear factor-κB; NOX: NADPH oxidase. The results are presented as the minimum, median, maximum, and percentiles. ns: not significant. * *p*  <  0.05.

Furthermore, NOX activity was significantly higher in the group of rats with DM1 compared with those treated with insulin. We also observed an upward trend in NF-κB activation in the skin of rats with DM1, although this result was not statistically significant (Figure 7).

### 3.6. The Effect of Insulin Therapy on Extracellular Matrix Remodeling in the Skin of Diabetic Rats

**Figure 8:**
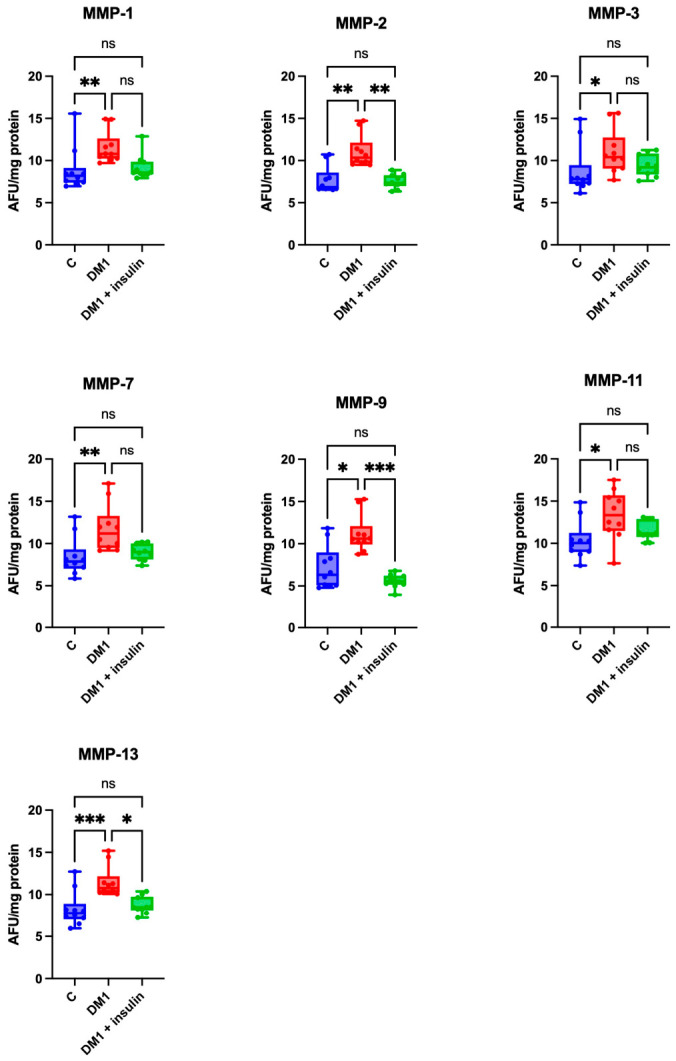
Matrix metalloproteinase activity in skin tissue homogenates in C (n = 10; blue bars), DM1 (n = 10; red bars), and DM1+insulin (n = 10; green bars) rats. C: control group; DM1: diabetic rat group; DM1+insulin: insulin-treated diabetic rat group; MMP-1: metalloproteinase-1; MMP-2: metalloproteinase-2; MMP-3: metalloproteinase-3; MMP-7: metalloproteinase-7; MMP-9: metalloproteinase-9; MMP-11: metalloproteinase-11; MMP-13: metalloproteinase-13. The results are presented as the minimum, median, maximum, and percentiles. ns: not significant. * *p*  <  0.05; ** *p* < 0.01; *** *p* < 0.001.

In this study, it was shown that MMP-1 activity was higher (32%) in the skin of DM1 group compared to the skin of control animals. However, MMP-2 activity in the skin of DM1 rats was higher (51%) than in the skin of control animals. Moreover, MMP-2 activity was significantly lower (−29%) in the skin of insulin-treated diabetic rats compared to DM1 group. MMP-3 activity was higher (34%) in the skin of DM1 rats compared to the control group. Similarly, MMP-7 activity was significantly elevated (42%) in the skin of DM1 group compared to control group (Figure 8).

MMP-9 activity was significantly higher (67%) in the skin of DM1 rats compared to the control group. Additionally, MMP-9 activity was significantly lower (−47%) in the skin of insulin-treated diabetic rats than in DM1 rats. MMP-11 activity in the skin of DM1 rats was higher (33%) than in control animals. Finally, MMP-13 activity was markedly higher (39%) in the skin of the DM1 group than in the control group, while its activity was significantly reduced (−21%) in the skin of insulin-treated diabetic rats compared to the DM1 group (Figure 8).

### 3.7. Correlations

ONOO- concentration in rat skin homogenates showed a positive correlation with Cas-3 activity (r = 0.802, *p* < 0.0001) and GSH-Px activity (r = 0.789, *p* < 0.0001). Furthermore, GSH concentration was positively correlated with GSH-Px activity (r = 0.748, *p* < 0.0001). A positive correlation was also observed between GSH concentration and MMP-2 activity (r = 0.727, *p* < 0.0001) and MMP-9 activity (r = 0.778, *p* < 0.0001). GSH concentration also positively correlated with GLU activity (r = 0.52, *p* < 0.01) in rat skin.

Correlation analysis showed a positive relationship between GSH-Px activity and the levels of DT (r = 0.919, *p* < 0.0001), NFKN (r = 0.804, *p* < 0.0001), and AGEs (r = 0.858, *p* < 0.0001) in rat skin. At the same time, GSH-Px activity showed a positive correlation with the activity of several MMPs: MMP-1 (r = 0.898, *p* < 0.0001), MMP-2 (r = 0.947, *p* < 0.0001), MMP-3 (r = 0.781, *p* < 0.0001), MMP-7 (r = 0.896, *p* < 0.0001), MMP-9 (r = 0.851, *p* < 0.0001), MMP-11 (r = 0.748, *p* < 0.0001), and MMP-13 (r = 0.945, *p* < 0.0001) in rat skin.

DT content was positively associated with NFKN (r = 0.894, *p* < 0.0001) and AGE (r = 0.957, *p* < 0.0001) content in rat skin. Furthermore, it was observed that DT content positively correlated with the activity of MMP-1 (r = 0.899, *p* < 0.0001), MMP-2 (r = 0.876, *p* < 0.0001), MMP-3 (r = 0.774, *p* < 0.0001), MMP-7 (r = 0.929, *p* < 0.0001), MMP-9 (r = 0.793, *p* < 0.0001), MMP-11 (r = 0.832, *p* < 0.0001), and MMP-13 (r = 0.955, *p* < 0.0001) in rat skin.

Further analysis revealed a positive correlation between KN content and NFKN (r = 0.888, *p* < 0.0001) and AGE (r = 0.829, *p* < 0.0001) levels in rat skin. A positive correlation was also observed between NFKN content and the activity of MMP-1 (r = 0.78, *p* < 0.0001), MMP-2 (r = 0.739, *p* < 0.0001), MMP-7 (r = 0.814, *p* < 0.0001), MMP-11 (r = 0.766, *p* < 0.0001), and MMP-13 (r = 0.841, *p* < 0.0001) in rat skin. Furthermore, it was shown that the AGE content correlated positively with the NFKN content (r = 0.964, *p* < 0.0001) in rat skin. In parallel, a positive correlation was demonstrated between AGE content and the activity of MMP-1 (r = 0.823, *p* < 0.0001), MMP-2 (r = 0.787, *p* < 0.0001), MMP-3 (r = 0.744, *p* < 0.0001), MMP-7 (r = 0.863, *p* < 0.0001), MMP-11 (r = 0.796, *p* < 0.0001) and MMP-13 (r = 0.888, *p* < 0.0001) in rat skin.

## 4. Discussion

**Figure 9:**
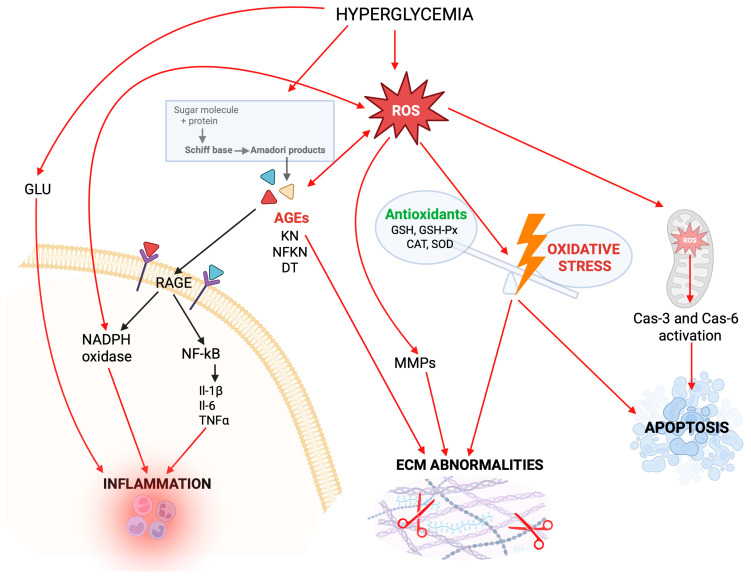
Relationship between oxidative stress, protein glycation, and extracellular matrix remodeling in diabetic skin. ROS: Reactive oxygen species; AGEs: Advanced glycation end products; KN: Kynurenine; NFKN: N-formylkynurenine; DT: Dityrosine; RAGE: Receptors for advanced glycation end products; NF-Κb: Nuclear factor-κB; TNFα: Tumor necrosis factor alpha; IL-1β: interleukin-1 beta; IL-6: interleukin-6; GLU: β-glucuronidase; MMPs: Matrix metalloproteinases; GSH: Reduced glutathione; GSH-Px: Glutathione peroxidase; CAT: Catalase; SOD: Superoxide dismutase; Cas-3: Caspase-3; Cas-6: Caspase-6; ECM: Extracellular matrix. Created in BioRender. Maciejczyk, M. (2026) https://BioRender.com/acykuw3 (accessed on 27 April 2026).

Hyperglycemia contributes to an imbalance between the production and removal of ROS and reactive nitrogen species (RNS) (Figure 9). The main sources of ROS and RNS are the mitochondrial electron transport chain (glucose increases the production of superoxide radicals), the auto-oxidation of glucose and overproduction of glycation products, including AGEs, increased activity of NADPH oxidase and endothelial nitric oxide synthase (eNOS), as well as activation of the polyol and hexosamine pathways [51,52,53,54,55]. Under normal physiological conditions, antioxidant compounds, such as GSH, SOD, CAT, and GSH-Px, break down ROS and RNS, playing a key role in reducing the effects of OS [9,56]. Our study revealed increased expression of non-enzymatic (↑ GSH) and enzymatic (↑ GSH-Px) antioxidants in the skin of DM1 rats (Figure 5). This may indicate a strengthening of the skin’s antioxidant capacity, which represents a compensatory response to increased ROS and RNS production in DM1 skin [57]. This hypothesis can be supported by a study investigating the activity of another endogenous antioxidant, CAT, in the epidermis and dermis of human skin during photoaging. The findings demonstrated that CAT activity is differentially regulated following acute (↓ CAT) and chronic (↑ CAT) UV exposure, both of which promote free radical generation [58]. Notably, skin affected by chronic diabetes also exhibits features of accelerated aging. In our research, only the thiol antioxidant barrier is enhanced. Antioxidants containing -SH groups are considered the most important for the skin. The GSH action involves the removal of hydrogen peroxide and organic peroxides, as well as the chelation of pro-oxidative metal ions. Therefore, in skin cells, GSH regulates repair processes by influencing keratinocyte proliferation and ECM remodeling [59]. Interestingly, we showed a positive correlation between GSH-Px activity and DT (r = 0.919, *p* < 0.0001), NFKN (r = 0.804, *p* < 0.0001) and AGE levels (r = 0.858, *p* < 0.0001), suggesting relationship between antioxidant defense and the formation of glycoxidation products. Many studies have shown higher skin autofluorescence (SAF) in DM1 patients and confirm a close association between the level of AGEs in skin collagen and the duration and severity of hyperglycemia [60,61,62]. It is well known that accumulation of AGEs in collagen fibers causes loss of skin elasticity and stiffness. Some skin complications of DM1, such as acquired reactive perforating collagenosis or scleroderma diabeticorum, result from thickening and hardening of the skin caused by AGE-induced degeneration of connective tissue [63]. In our model, an increase in the accumulation of glycation (↑ AGEs) and glycooxidation (↑ DT, ↑ KN, ↑ NFKN) products was observed in the skin of DM1 rats (Figure 6). Elevated levels of KN and NFKN may provide insight into the progression of degenerative processes in DM1 skin tissue [64,65]. Kynurenine metabolites may serve as valuable markers of glycation and inflammation in the skin [66]. Increased KN expression activates inflammatory signaling, accompanied by an increase in OS levels [65,67].

The primary receptor for AGEs and other glycoxidation products is RAGE [68]. It was shown that the AGE–RAGE interaction induces ROS generation via NOX activation. The NOX enzyme family comprises several members (NOX1-NOX5), and their primary role is to catalyze the production of superoxide radicals by reducing NADPH. Elevated blood glucose triggers NOX enzymes to overproduce ROS, disrupting redox homeostasis and resulting in OS, low-grade inflammation, and cellular and tissue damage [69,70]. AGE–RAGE interaction also activates downstream signaling pathways, including the NF-κB transcription factor [71]. Activated NF-κB translocates to the nucleus, where it enhances the expression of numerous inflammatory mediators, including tumor necrosis factor alpha (TNF-α), interleukin-1 beta (IL-1β), and interleukin-6 (IL-6), as well as apoptosis-related genes, thereby promoting inflammation, apoptosis, and tissue damage [71,72]. In our study, we did not assess RAGE expression, but rather its downstream signaling, and we demonstrated increased NOX activity and an upward trend in NF-κB activation (Figure 7). This can be supported by other studies showing that DM1 is associated with enhanced and prolonged skin inflammation, as evidenced by increased numbers of inflammatory cells, including neutrophils [73]. Excessive neutrophil activation may result in sustained release of inflammatory cytokines, uncontrolled oxidative damage and cell death [74]. In our study, we evaluated the inflammatory marker GLU, a lysosomal enzyme that accumulates in neutrophil primary granules (Figure 4). Elevated GLU activity in the skin may indicate increased neutrophil influx during inflammation [75]. However, we evaluated only a single biomarker, so further studies are needed to assess cytokines, chemokines, and growth factors in order to fully characterize the inflammatory profile in diabetic skin.

In DM1, metabolic disorders are another key factor contributing to the development of OS [76]. Serious systemic metabolic disorders occur as a consequence of glycosuria (the presence of glucose in the urine), which leads to frequent urination and fluid loss, in turn causing polyphagia (increased appetite) and polydipsia (increased thirst) [77]. Both polyphagia and polydipsia reflect the body’s difficulty in utilizing glucose, leading to dehydration and cellular malnutrition, thereby disrupting cellular metabolism and redox homeostasis [76].

Individuals with DM1 frequently exhibit features of accelerated cutaneous aging [78]. Histological analyses have demonstrated a deterioration of the epidermis’ multilayered architecture, accompanied by abnormalities in keratinocyte proliferation that contribute to epidermal thinning. Furthermore, histopathological examination has revealed disruption of collagen organization and the presence of inflammatory cell infiltrates. This form of endogenous tissue damage occurs in clinically intact DM skin and is primarily driven by chronic hyperglycemia, which promotes disturbances in ECM remodeling and persistent inflammatory processes [71,73,79,80,81,82]. In our study, we found that several MMPs, including MMP-1, -2, -3, -7, -9, -11, and -13, are significantly elevated in skin tissue of DM1 rats, thereby affecting collagen remodeling and leading to ECM disruption (Figure 8). Our study also revealed a statistically significant association between AGE concentrations and elevated levels of MMP-1 (r = 0.823, *p* < 0.0001) and MMP-2 (r = 0.787, *p* < 0.0001). We also observed a positive correlation between DT concentration and the levels of NFKN (r = 0.894, *p* < 0.0001), AGE (r = 0.957, *p* < 0.0001), and MMP-1 (r = 0.899, *p* < 0.0001), MMP-2 (r = 0.876, *p* < 0.0001), MMP-3 (r = 0.774, *p* < 0.0001), MMP-7 (r = 0.929, *p* < 0.0001), MMP-9 (r = 0.793, *p* < 0.0001), MMP-11 (r = 0.832, *p* < 0.0001) and MMP-13 (r = 0.955, *p* < 0.0001). This observation is consistent with previous studies linking glycated proteins, OS and DM1-associated inflammation with increased stiffness of the reticular dermis caused by changes in the composition and organization of matrix proteins [83,84,85,86]. In a high glucose environment, skin tissue produces excessive amounts of ROS, which activates MMPs and leads to increased ECM remodeling [63,86]. Data suggest that delayed wound healing in DM1 results from AGE-dependent modulation of MMP-9 expression in keratinocytes, which disrupts epithelial remodeling [87,88,89,90].

Of all RNS, ONOO- is a highly active oxidant inducing apoptosis in various cell types [91]. The primary target of ONOO- is the vascular endothelium, which explains the increased concentration of this compound in vascular pathologies associated with DM1 [91,92]. Apoptosis occurs when the accumulation of ONOO- exceeds the capacity of the endogenous antioxidant defense system and DNA repair mechanisms. In the case of the skin, keratinocytes undergo apoptosis because of excessive production of ROS and RNS and the accumulation of ONOO- molecules [51]. Cas-3 and Cas-6 are enzymes involved in the induction and execution phases of apoptosis [51,93]. Studies of human pancreatic tissue in DM1 have shown that β-cells undergo apoptosis significantly more often than in patients without DM1, and Cas-3 activation can be triggered by metabolic stress, which plays a key role in the pathophysiology of DM1 [93,94,95,96,97]. Hyperglycemia, along with increased AGE and RAGE expression, arrests the cell cycle and induces apoptosis in dermal fibroblasts [98]. In our study, increased activity of Cas-3 and Cas-6 in DM1 skin may result from the intense ROS and RNS production in response to high glucose concentrations (Figure 3). We observed a statistically significant correlation between ONOO- concentration and Cas-3 activity (r = 0.802, *p* < 0.0001), which may confirm the activation of apoptotic pathways in DM1 skin cells.

Insulin therapy is a cornerstone of treating DM1, replacing the body’s missing insulin to regulate blood sugar levels. In our study, insulin administration reduced apoptosis in skin cells (Figure 3) and improves the antioxidant status of the skin, particularly glutathione system (↑ GSH and ↑ GSH-Px) (Figure 5). Following insulin therapy, production of glycation and glycoxidation products (Figure 6), as well as MMP levels (Figure 8), is also reduced. The observed changes may result from an improvement in the metabolic status of DM1 rats—it is well known that maintaining proper glucose levels is the primary mechanism protecting against the development of diabetic complications. A growing body of literature also points to the role of local insulin injection in tissue regeneration and wound healing by accelerating the proliferation and migration of keratinocytes, endothelial cells, and fibroblasts [99,100]. Topical insulin is a promising treatment for slow-healing wounds in DM1. Results of efficacy studies in rats with DM1 using an insulin cream showed that insulin stimulates keratinocyte migration, leading to re-epithelialization and contributing to the formation of a new, functional epidermal layer [99,101,102]. Insulin therapy also increased the expression of proteins in insulin signaling pathways, such as AKT, which enhances protein synthesis and vascular endothelial growth factor (VEGF) release in skin wounds, thereby promoting the mobilization and migration of endothelial progenitor cells to the site of injury [99,101]. Enhanced activity of MMPs, especially MMP-9 is associated with impaired wound healing [103]. Contrary to earlier reports, some studies suggest that the anabolic effects of insulin therapy are not sufficient to improve neutrophil function and lead to a complete reduction in MMP-9 activity, thereby accelerating wound healing. This may partly explain why MMP activity did not return to the levels observed in the control group following insulin administration. Additionally, it has been shown that insulin stimulates macrophages, which secrete growth factors and anti-inflammatory cytokines reducing inflammation in the body [104,105]. ONOO- concentration, NOX expression and Cas-3 activity decreased significantly following insulin administration, which may indicate insulin’s anti-apoptotic properties through caspase inhibition and reduced ROS and RNS production (Figure 4) [106,107,108]. However, despite insulin treatment, skin inflammation persisted, which may indicate a long-term effect of cytokines and AGEs on skin tissue. Both inflammation and glycation are chronic processes that accumulate over time. Glycation slowly and irreversibly damages collagen, triggering chronic, long-term inflammatory pathways in the skin [13].

Further research is needed to assess the parameters of OS, protein glycation, and ECM remodeling in diabetic skin. Our animal model relates only to DM1 and assesses just selected antioxidants and markers of protein glycation, ECM degradation, and apoptotic factors. Correlating biomarker information with histological assessment of skin lesions would provide more valuable insights and a better understanding of the significance of the complexity of these processes. There is an urgent need to expand future research on skin parameters to include molecular studies of both DM1 and DM2 to better understand disease pathogenesis and enable early detection. However, the greatest diagnostic significance would come from expanding the studies from limited, controlled samples to a broader, more representative patient population. With the global increase in the prevalence of DM1, clinical trials have become a fundamental part of medical research. A precise, detailed explanation of the pathophysiological and immunological processes underlying skin changes that may be an early indicator of DM1 could significantly reduce complications and mortality in the future.

## 5. Conclusions

1. In rats with DM1, an enhanced thiol antioxidant defense is observed, whilst other antioxidant enzymes show no response. The result is increased oxidative stress in the skin.
2. In the skin of DM1 rats, we observe increased protein glycation and glycoxidation, as well as enhanced extracellular matrix (ECM) remodeling.
3. Strong correlations between the antioxidant systems, products of glycation and glycoxidation, and MMP activity may suggest a possible link between redox homeostasis and the remodeling of the skin’s ECM.
4. Insulin treatment normalizes the skin’s antioxidant barrier and diminishes oxidative stress. It also reduces the intensity of protein glycation and glycoxidation, though not to the levels observed in the control group.
5. The effects of insulin therapy are not sufficient to completely reduce MMP activity.
6. DM1 enhances peroxynitrite-induced apoptosis, but insulin restores physiological balance in skin cells.

**Figure 10:**
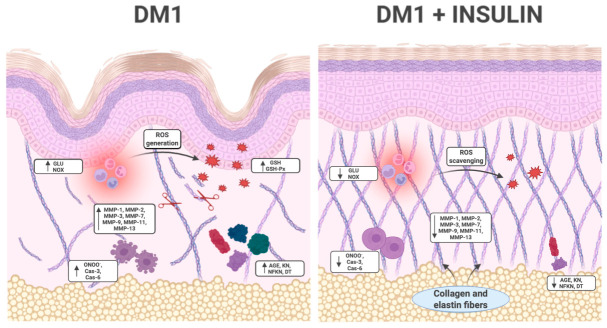
Graphical conclusions from the study. DM1: diabetic rat group; DM1+insulin: insulin-treated diabetic rat group; ROS: Reactive oxygen species; AGE: Advanced glycation end products; KN: Kynurenine; NFKN: N-formylkynurenine; DT: Dityrosine; GLU: β-glucuronidase; NOX: NADPH oxidase; MMP-1: Matrix metalloproteinases-1; MMP-2: Matrix metalloproteinases-2; MMP-3: Matrix metalloproteinases-3; MMP-7: Matrix metalloproteinases-7; MMP-9: Matrix metalloproteinases-9; MMP-11: Matrix metalloproteinases-11; MMP-13: Matrix metalloproteinases-13; GSH: Reduced glutathione; GSH-Px: Glutathione peroxidase; ONOO-: Peroxynitrite; Cas-3: Caspase-3; Cas-6: Caspase-6. Created in BioRender. Maciejczyk, M. (2026) https://BioRender.com/acykuw3 (accessed on 27 April 2026).

The study’s conclusions are presented graphically in Figure 10.

## Data Availability

The data presented in this study are available on request from the corresponding author due to privacy restrictions or ethical reasons.

## Abbreviations

The following abbreviations are used in this manuscript:

DM1	Type 1 diabetes
ROS	Reactive oxygen species
AGEs	Advanced glycation end products
RAGE	Receptors for advanced glycation end products
MAPK	Mitogen-activated protein kinase
PKC	Protein kinase C
ECM	Extracellular matrix
MMPs	Matrix metalloproteinases
OS	Oxidative stress
EMC	Experimental Medicine Centre
STZ	Streptozotocin
BCA	Bicinchoninic acid
ONOO-	Peroxynitrite
SOD	Superoxide dismutase
Cas-3	Caspase-3
Cas-6	Caspase-6
pNA	p-nitroaniline
HEX	N-acetyl-β-hexosaminidase
GLU	β-glucuronidase
GSH	Reduced glutathione
GSH-Px	Glutathione peroxidase
DTNB	5.5′-dithiobis-(2-nitrobenzoic acid)
NADPH	Nicotinamide adenine dinucleotide
NADP^+^	Nicotinamide adenine dinucleotide phosphate
CAT	Catalase
AFU	Fluorescence units
DT	Dityrosine
KN	Kynurenine
NFKN	N-formylkynurenine
TYR	Tyrosine
APMA	4-Aminophenylmercuric Acetate
MCA	7-methoxycoumarin-4-yl)acetate
RNS	Reactive nitrogen species
eNOS	Endothelial nitric oxide synthase
NF-κB	Nuclear factor-κB
TNF-α	Tumor necrosis factor alpha
IL-1β	Interleukin-1 beta
IL-6	Interleukin-6
VEGF	Vascular Endothelial Growth Factor
